# The efficacy of targeted therapy combined with radiotherapy and temozolomide-based chemotherapy in the treatment of glioma: A systemic review and meta-analysis of phase II/III randomized controlled trials

**DOI:** 10.3389/fonc.2023.1082539

**Published:** 2023-01-26

**Authors:** Yifan Ma, Yue Wang, Chen Nie, Yongzhong Lin

**Affiliations:** ^1^ Department of Neurology, The Second Affiliated Hospital of Dalian Medical University, Dalian, Liaoning, China; ^2^ Department of Medical Oncology, The Second Affiliated Hospital of Dalian Medical University, Dalian, Liaoning, China

**Keywords:** targeted therapy, radiotherapy, glioma, chemotherapy, temozolomide

## Abstract

**Background:**

Glioma is the most common intracranial tumor, accounting for about half of the primary intracranial tumors, with the characteristics of hidden onset and high mortality. Even after surgery, radiotherapy and chemotherapy, the prognosis of glioma is not ideal. Targeted therapy has developed rapidly in the treatment of other malignant tumors, which is also an important direction in the research and development of new therapies for glioma. So far, targeting combined with radiotherapy and chemotherapy have been used as the treatment of glioma in many clinical trials, but the role of targeted combined radiotherapy and chemotherapy in the treatment of glioma is still controversial. The purpose of this study was to evaluate the efficacy of targeted therapy combined with radiotherapy and temozolomide (TMZ)-based chemotherapy in the treatment of glioma.

**Methods:**

Phase II or phase III clinical trials involving targeted therapy combined with radiotherapy and chemotherapy and temozolomide-based radiotherapy and chemotherapy for gliomas were searched using PubMed, Embase and Web of Science databases, and a comprehensive meta-analysis was conducted. The primary outcome was overall survival time (OS) and progression-free survival time (PFS), and the secondary outcome was adverse reaction. The time-to-event data is summarized as hazard ratio (HR), and the binary results are summarized as odds ratio (OR). Two researchers conducted literature screening, data extraction and quality evaluation according to inclusion and exclusion criteria. Stata16.0 software was used for analysis, random effect model was used for data merging, and forest map was used for display.

**Results:**

A total of 11 eligible literatures and 12 prospective randomized controlled clinical trials of 1284 cases were included in the meta-analysis. The results showed that compared with radiotherapy and chemotherapy alone, targeted drugs combined with temozolomide-based radiotherapy and chemotherapy could significantly improve OS in phase II trial, but there was no improvement in Phase III trial, and PFS of newly diagnosed glioma patients was improved (HR=0.82(0.71-0.94) 95%CI, *p* =0.005). The PFS of the third phase of the experiment also improved. Compared with radiotherapy and chemotherapy alone, there was no statistically significant increase in adverse events in targeted combined radiotherapy and chemotherapy group.

**Systematic review registration:**

https://www.crd.york.ac.uk/prospero, identifier CRD42022326012.

## 1 Introduction

Glioma (GM) is the most common primary malignant brain tumor, which can occur anywhere in the central nervous system, but mainly in the brain and glial tissue (1). Glioblastoma(GBM), the most common glioma histology, accounts for 60-70% of all gliomas (2), which is the most malignant tumor (World Health Organization grade IV) and associated with a poor prognosis (3). The standard treatment of glioblastoma includes the largest range of surgical resection, radiotherapy and alkylation chemotherapy. However, due to the invasiveness of this disease, complete resection is almost impossible and recurrence is almost inevitable (4). Postoperative concurrent radiotherapy and chemotherapy is the standard treatment. The most common chemotherapy drug used for treatment is temozolomide (TMZ), an alkylating agent that sensitizes cells to radiation (3). Studies have shown that TMZ treatment and radiotherapy can improve OS for up to 15.7 months (5). Nevertheless, the prognosis of these patients is poor, and the survival rate of more than 5 years is still unpleasant (about 5%) (6). The Cancer Genome Atlas (TCGA) is a government funded initiative aimed at classifying and identifying genomic changes in cancer pathogenesis. GBM is the first tumor with comprehensive molecular characterization. Three core pathways leading to the development of GBM have been identified:(i) receptor tyrosine kinase (RTK)/Ras/phosphoinositide 3-kinase (PI3K), (ii) p53, and (iii) retinoblastoma (Rb) pathway (7). In recent years, with the development of molecular targeted therapy or precision medicine, targeted therapies aim to inhibit specific molecular targets that lead to enhanced tumor growth (8). Targeted therapy has shown satisfactory results in a variety of cancers, including breast cancer, ovarian cancer, lung cancer (9–11), and these molecular targeted therapies are also promising in glioma. Antiangiogenic drugs are the most advanced molecular targeted therapies, and promising results have been observed in patients with recurrent glioma (12). Other molecular targeted therapies are currently undergoing preclinical or clinical evaluation, but published results from some of these trials do not show the expected therapeutic effects (13, 14). Therefore, there are still many controversies about the targeted combination therapy of radiotherapy and chemotherapy for glioma. In this study, we conducted a systematic review and meta-analysis of randomized controlled trials (RCTs) to evaluate the efficacy of targeted therapy combined with TMZ based chemotherapy and radiotherapy for glioma.

## 2 Materials and methods

### 2.1 Register

In this systematic review and meta-analysis, targeted combined radiotherapy and chemotherapy in gliomas were compared with temozolomide-based chemotherapy and radiotherapy to evaluate the efficacy and safety of targeted combined radiotherapy and chemotherapy. The report is based on the recommendations of the Preferred Reporting Project for System Review and Meta-analysis (PRISMA). The system review has been prospectively registered in PROSPERO (CRD42022326012).

### 2.2 Search strategy

The English literatures about targeted therapy combined with radiotherapy and chemotherapy and temozolomide-based radiotherapy and chemotherapy for glioma published from inception to October 2022 were searched in PubMed, Embase and Web of Science databases, and related clinical trials were also searched in the Clinical trial Registry (https://www.clinicaltrials.gov/). The search keywords are as follows: glioma, chemotherapy, retrieved of all phase II or phase III prospective randomized controlled clinical trials and including appropriate data for analysis. If the article meets the research criteria, the full text will be retrieved. If there are duplications (patient data from the same trial or institution), try to select the most complete, up-to-date and relevant study.

### 2.3 Trials selection

The two authors (Ma and Wang) independently screened the qualifications of all the identified references. Any differences will be resolved through discussion and consultation. The flow chart records the selection process of the experiment and gives the specific reasons for excluding the study at each stage. We limited the search to randomized controlled clinical trials that comparing targeted combination radiotherapy and chemotherapy with temozolomide alone. Trials that did not contain temozolomide in evaluation of vaccine therapy and any other combination or radiotherapy and chemotherapy regimens were excluded. Clinical trials meeting all of the following inclusion criteria met the criteria: patients with newly diagnosed or recurrent gliomas who received radiotherapy and chemotherapy with temozolomide; the main outcome indicators (at least OS) and Kaplan-Meier (KM) survival curve were provided. The exclusion criteria were non-prospective studies; non-randomized controlled trials; single-arm trials; animal studies; simple drug dose studies; letters, reviews and editorials; and publications that did not provide major outcome indicators and were unpublished or unable to retrieve the full text. If there were multiple publications in a single clinical trial, all publications were included and the results were complementary, but the longest follow-up period is preferred.

### 2.4 Quality assessment

**Figure 1:**
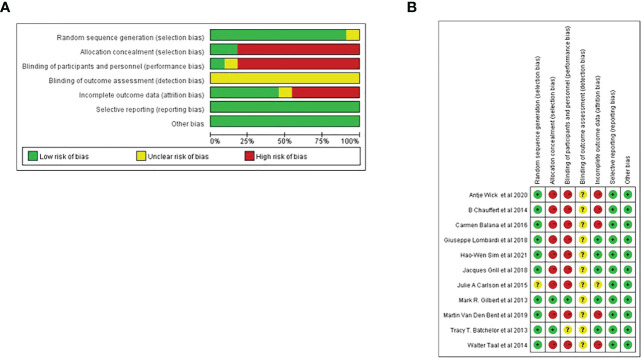
Risk of bias. **(A)** Risk of bias graph. **(B)** Risk of bias summary.

Cochrane bias risk assessment tool was used to analyze the bias risk of the included randomized double-arm trial. Each of the following areas was evaluated at the trial level: hidden random sequence generation and allocation (selection bias); blind method of participants and personnel (performance bias); blind method of outcome evaluation (test bias); incomplete result data (attrition bias); selective results report (report bias). And other deviations (for example, baseline imbalance, early termination of trials, industry or funding deviations, missing sample size calculations or other defects in statistical analysis). Each potential source of deviation is rated as “high”, “low” or “unclear” risk (**Figure 1**).

### 2.5 Data extraction

Two researchers independently searched the literature and extracted the data. If there is any dispute, it will be discussed and resolved with a third party. The extracted research data include the author’s name, publication year, trial stage, type of tumor, patient demographic statistics and treatment methods. The observation indexes included 6-month OS, 12-month OS, 6-month PFS, 12-month PFS, median survival time, median progression-free survival time, and rate of adverse events ≥ grade 3. If direct data is not provided directly in this paper, the overall survival rate and progression-free survival rate are estimated by Kaplan-Meier diagram. The primary endpoints were overall survival and progression-free survival (if progression-free survival was not available, disease-free survival was used). The secondary end point was the occurrence of adverse events, which were classified according to toxicity≥ 3, and the study included leukopenia, neutropenia, thrombocytopenia, lymphocytopenia, nervous system (mainly headache).

### 2.6 Statistical analysis

The hazard ratio (HR) and their respective 95% confidence intervals (CI) were evaluated as a measure of the effectiveness of the time-to-event data. If the study reports adjusted and unadjusted hazard ratios, the adjusted hazard ratios are used for primary analysis. If the hazard ratio is not reported but there is sufficient information (e.g., Kaplan-Meier diagram), the estimation method described by Tierney is applied to estimate the hazard ratio and their respective 95% confidence intervals (15). For the binary classification results, the odds ratio (OR) and its respective 95% confidence intervals are regarded as effects. Stata16.0 was used for meta-analysis, and forest maps were drawn for analysis. I^2^ statistics were used for heterogeneity test. If there is no significant heterogeneity between studies (I^2^ ≤ 50%, *p* < 0.05), the fixed effect model was used to merge the data. If there is significant heterogeneity between studies (I^2^ > 50%, *p*≥ 0.05), random effects model was used to merge the data. The heterogeneity was studied by subgroup analysis and meta regression analysis. Publication bias was evaluated by funnel chart and Egger test.

## 3 Result

### 3.1 Eligible studies

**Figure 2:**
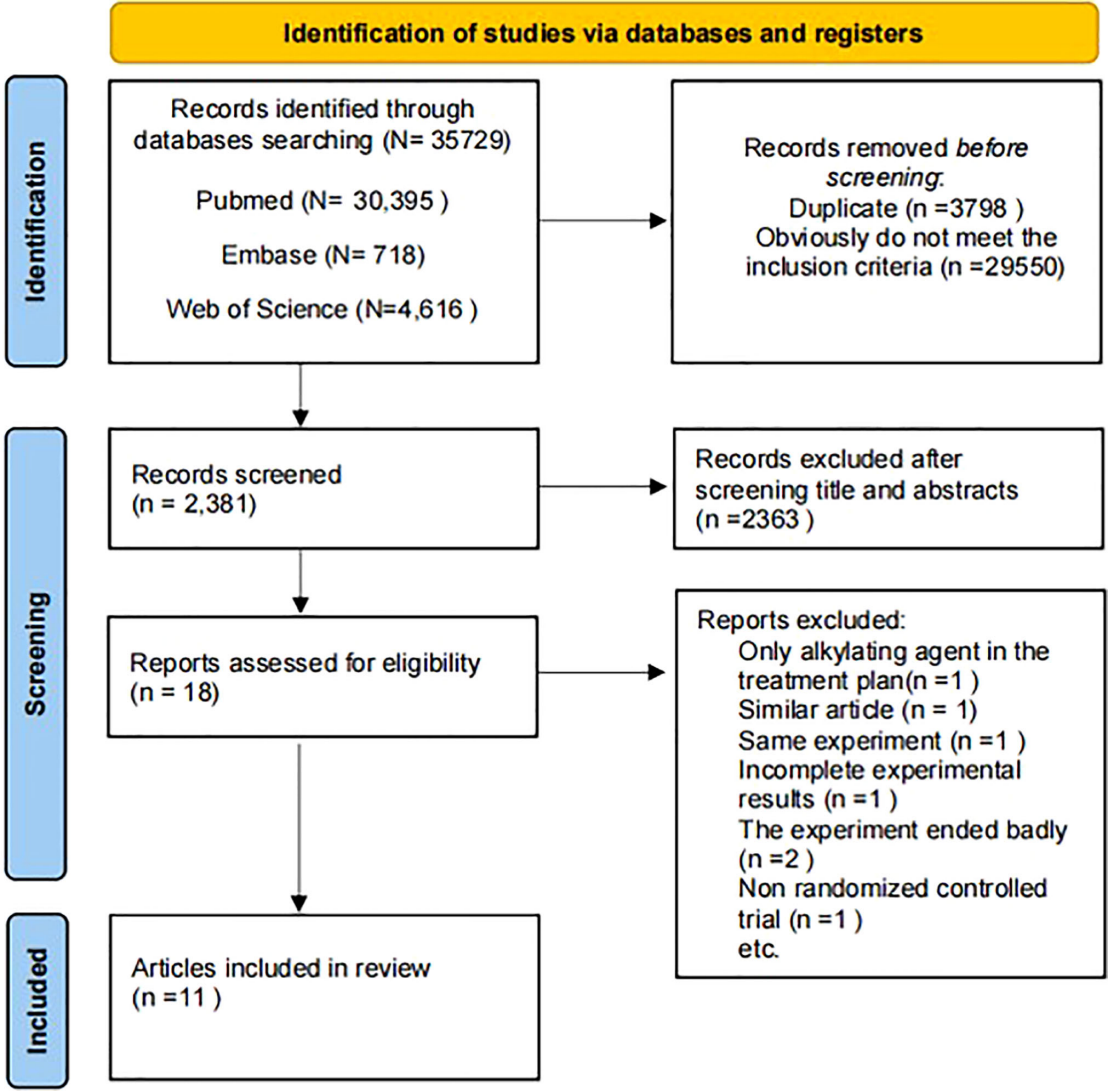
Study diagram.

According to the retrieval method mentioned above, a total of 35729 potentially relevant studies were assessed. The detailed steps of the search are shown in **Figure 2**. After the selection procedure, eleven articles were included (13, 16–25), with a total of 1284 patients with glioma (715 patients receiving the targeted drug therapy combined with radiotherapy and chemotherapy arm and 569 patients receiving the Simple radiotherapy and chemotherapy). The basic characteristics of these studies were showed in **Table 1**.

**Table 1:** Basic characteristics of the studies included in this meta-analysis.

Include studies	Publish time	Study patients	Experiment type	Experiment phase	Number of participants		Median age (range)		Male		Interventions			Primary Outcome	Secondary outcome
					Experimental group	Control group	Experimental group	Control group	Experimental group	Control group	Experimental group	Control group			
Antje Wick	2020	Grade III and IV malignant glioma	RCT	II	40	16	58.7 (8.9)*	57.8 (11.6)	22 (55.0)	11 (68.8)	Galunisertib + TMZ/RT	TMZ/RT		PFS OS	AE above grade 3 (neutropenia, leukopenia, thrombocytopenia, lymphocytopenia, gastrointestinal discomfort, headache)
Carmen Balana	2016	glioblastoma	RCT	II	48	45	62.9(43-75)	62(36-75)	31 (64.6)	25 (55.6)	TMZ 85 mg/ 75 mg+RT60 Gy+ BEV10 mg	TMZ/RT		PFS OS	AE above grade 3 (neutropenia, leukopenia, thrombocytopenia, lymphocytopenia, gastrointestinal discomfort, headache)
Julie A Carlson	2015	GBM	RCT	II	30	26	56.5(31–78)	60.5(25–77)	17	16	RT 60 Gy /30 Gy+TMZ75 mg+Adjuvant TMZ150–200 mg+Bev 10 mg	RT/TMZ		PFS OS	NA
Jacques Grill	2018	Grade III and IV glioma	RCT	II	62	59	10.0 (3-17)	11.0 (3-17)	34 (55)	36 (61)	RT/TMZ+BEV10mg	RT/TMZ		PFS OS	NA
B Chauffert	2014	GB	RCT	II	60	60	60.2 (43–69)	60.9 (43–71)	34 (56.7)	37 (61.7)	RT60 Gy/TMZ75 mg+Bev10 mg+IRI125 mg	RT/TMZ		PFS OS	AE above grade 3 (neutropenia, leukopenia, thrombocytopenia, lymphocytopenia, gastrointestinal discomfort, headache)
Martin Van Den Bent	2019	glioblastoma EGFR amplification	RCT	II	88	86	59.2(40.1–75.4)	58.8 ( 34.9–82.3)	59	58	RT/TMZ + ABT-414	RT/TMZ		PFS OS	AE above grade 3 (neutropenia, leukopenia, thrombocytopenia, lymphocytopenia, gastrointestinal discomfort, headache)
Mark R. Gilbert	2013	glioblastoma	RCT	III	312	309	<50 57 (18%) ≥50 255 (82%)	<50 65 (21%) ≥50 244 (79%)	178	194	bevacizumab +RT/TMZ	RT/TMZ		PFS OS	AE above grade 3 (neutropenia, thrombocytopenia, lymphocytopenia, gastrointestinal discomfort, headache)
Tracy T. Batchelor	2013	recurrent glioblastoma	RCT	III	129	65	54	54	NA	NA	Cediranib + Lomustine +RT/TMZ	Lomustine + RT/TMZ+pla		PFS OS	AE above grade 3 (neutropenia, leukopenia, thrombocytopenia, lymphocytopenia, gastrointestinal discomfort, headache)
Tracy T. Batchelor	2013	recurrent glioblastoma	RCT	III	131	65	54	54	NA	NA	Cediranib+RT/TMZ	Lomustine + RT/TMZ+pla		PFS OS	AE above grade 3 (neutropenia, leukopenia, thrombocytopenia, lymphocytopenia, gastrointestinal discomfort, headache)
Hao-Wen Sim	2021	glioblastoma with unmethylated *MGMT* promoter region	RCT	II	84	41	60(22-78)	62(24-73)	59	28	veliparib+RT/TMZ	RT/TMZ		PFS OS	AE above grade 3 (neutropenia, thrombocytopenia, lymphocytopenia, gastrointestinal discomfort, headache)
Walter Taal	2014	recurrent glioblastoma	RCT	II	44	46	58 (24–73)	58 (37–77)	30	32	BEV/LOM+RT/TMZ	LOM+RT/TMZ		PFS OS	Grade 3 or above AE (leukopenia, thrombocytopenia)
Giuseppe Lombardi	2018	recurrent glioblastoma	RCT	II	59	60	54.8 (46.8–61.3)	58.9 (51.8–65.2)	41	43	Regorafenib+RT/TMZ	RT/TMZ+LOM		PFS OS	AE above grade 3 (neutropenia, thrombocytopenia, lymphocytopenia)

NA, Not Available.

### 3.2 Main results

**Figure 3:**
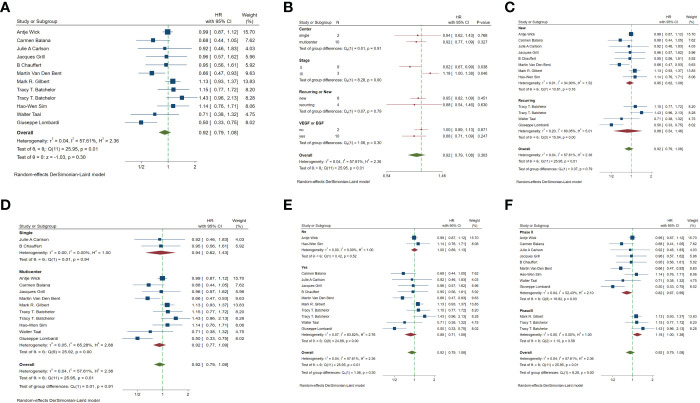
Forest plots of the included trials (OS) **(A)** Hazard ratio (HR) for overall survival (OS) of the included trials. **(B)** Subgroup analysis of OS. **(C)** Subgroups of patients with newly diagnosed and recurrent gliomas. **(D)** Subgroups of single-center and multi-center studies. **(E)** Subgroups of VEGF receptor inhibitors and EGF receptor inhibitors. **(F)** Subgroups of Phase II and phase III clinical trials.

**Figure 4:**
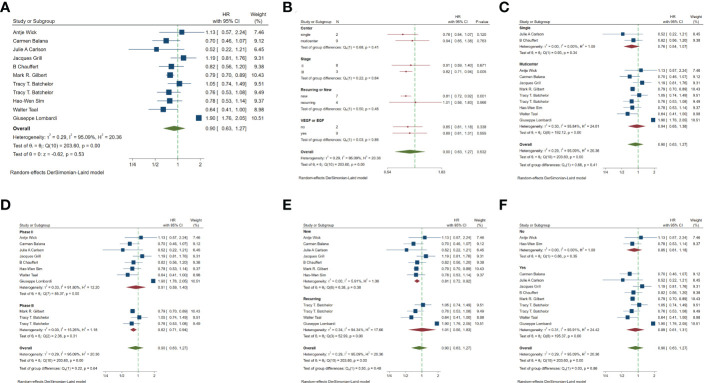
Forest plots of the included trials(PFS) **(A)** Hazard ratio (HR) for Progression-free survival (PFS) of the included trials. **(B)** Subgroup analysis of PFS. **(C)** Subgroups of single-center and multi-center studies. **(D)** Subgroups of Phase II and phase III clinical trials. **(E)** Subgroups of patients with newly diagnosed and recurrent gliomas. **(F)** Subgroups of VEGF receptor inhibitors and EGF receptor inhibitors.

In the included studies, 12 clinical trials reported OS, I^2 ^= 57.61%, *p*=0.01, and random effects model was used for meta-analysis. The results showed that the HR of OS in patients with targeted combined radiotherapy and chemotherapy was not statistically significant, HR=0.92 (0.79-1.08, 95% CI) (*p*=0.30) (**Figure 3A**). Therefore, we conducted a subgroup analysis of the included study (**Figure 3B**). The subjects of 8 trials were newly diagnosed patients, and 4 were patients with recurrent GM. The HR in the newly diagnosed patients is 0.95(0.82-1.09)(95%CI, *p*=0.451). The HR of patients with recurrent GM is 0.88 (0.54-1.46) (95% CI, *p*=0.630). Both of them showed no statistical significance. (**Figure 3C**). Two trials were single-center trials, and 10 trials were multi-center trials. In single-center trials, HR=0.94 (0.62-1.43), *p*=0.768; in multi-center trials, HR=0.92 (0.77-1.09), *p*=0.327, there was no statistical significance (**Figure 3D**). No VEGF or EFGR inhibitors were used in 2 trials, and VEGF or EFGR inhibitors were used in 10 trials. In the drug trials without VEGF or EFGR inhibitors, HR=1.00 (0.89-1.13) and there was no statistical significance (*p*= 0.971). In the drug trials using VEGF or EFGR inhibitors, HR=0.88 (0.71-1.09) showed no statistical significance (*p*=0.247) (**Figure 3E**). There were 9 phase II trials and 3 phase III trials. In the phase II trial, HR=0.82 (0.67-0.99), *p*=0.038. We found that compared with the radiotherapy and chemotherapy group, the OS of the radiotherapy and chemotherapy targeted combination group was significantly improved. In the phase III trial, HR=1.18 (1.00-1.38), p=0.046, there was no significant improvement in OS, the difference was statistically significant (**Figure 3F**). PFS was reported in 11 clinical trials using a random effect model, with I^2 ^= 95.09%, *p*<0.001. The results showed that HR=0.90 (0.63-1.27) and there was no statistical significance(*p*=0.53) (**Figures 4A** **, B**). Two single-center trials, HR=0.76 (0.54-1.07), showed no statistical significance (*p*=0.120); multi-center trials 9, HR=0.94 (0.65-1.38), and there was no statistical significance (*p*=0.763) (**Figure 4C**). There was no statistical significance in 8 phase II trials, HR=0.91 (0.59-1.40), *p*=0.671; phase III trials, HR=0.82 (0.71-0.94), *p*=0.005, respectively (**Figure 4D**). Seven tests of newly diagnosed patients, HR= 0.81 (0.72-0.92) 95% CI, *p*=0.001, indicating statistically significant improvement in PFS; for 4 trials in patients with recurrent GM, HR=1.01(0.56-1.83) 95% CI, *p*=0.966, there was no statistical significance (**Figure 4E**). There were 2 trials without VEGF or EFGR inhibitors, HR=0.85 (0.61-1.18, *p*=0.338) and 9 trials with VEGF or EFGR inhibitors, HR=0.89 (0.61-1.31, *p*=0.555) had no statistical significance (**Figure 4F**). The other endings are shown in **Table 2**.

**Table 2:** Display of other endings.

Include studies	Publish time	Median PFS (range)		PFS 6month (%)		PFS 12month (%)		OS median (range)		OS 6month (%)		OS 12month (%)	
		Experimental group	Control group	Experimental group	Control group	Experimental group	Control group	Experimental group	Control group	Experimental group	Control group	Experimental group	Control group
Antje Wick	2020	7.6 (6.1-10.4)	11.5 (5.4-15.9)	70	80	25 (14, 37)	47 (25, 66)	18.2 (13.4-20.6)	17.9 (10.7-24.0)	97	93	74 (59, 83)	80 (56, 92)
Carmen Balana	2016	4.8 (4.0-5.6)	2.2 (2.0-2.5)	40	20	6	9	10.6 (6.9–14.3)	7.7 ( 5.4–10.0)	77	57	48	36
Julie A Carlson	2015	12.8	9.4	80	85	66	38	16.3	16.3	94	93	76	65
Jacques Grill	2018	8.2 (7.8 -12.7 )	11.8 (7.9 -16.4)	68	66	39	49	16.2 ( 0,45.7 )	15.2 (0.1 ,46.8)	92	97	75	69
B Chauffert	2014	7.1 (5.5,9.2)	5.2 (4.3,6.8)	61.7	41.7	30	18	11.1 (9.0,15.0)	11.1 (9.0,15.0)	72	73	46	51
Martin Van Den Bent	2019	2.7 (2.0, 3.8)	1.9 (1.8, 2.0)	NA	NA	NA	NA	9.6 (7.4, 11.8)	8.2 (5.9, 9.5)	74	60	39.7 (29.4, 49.7)	28.2 (19.1, 37.9)
Mark R. Gilbert	2013	10.7 (10.0 -12.2)	7.3 (5.9 -7.9)	80	55	45	32	15.7 (14.2 -16.8)	16.1 (14.8 -18.7)	88	86	68	66
Tracy T. Batchelor	2013	4.2	2.7	27	24	5	14	9.4	9.8	76	70	35	41
Tracy T. Batchelor	2013	3	2.7	12	24	3	14	8	9.8	56	70	30	41
Hao-Wen Sim	2021	5.7 (3.9-6.5)	4.2 (2.4-5.7)	46 ( 36-57)	31 (18-46)	8	3	12.7 (11.4-14.5 )	12.8 (9.5-15.8)	82	90	56	53
Walter Taal	2014	4 (3–8)	1 (1–3)	41% (26–55)	13% (5–24)	22	2	11 (8–12)	8 (6–11)	79	64	45 (30–59)	30 (18–44)
Giuseppe Lombardi	2018	2.0 (1.9–3.6)	1.9 (1.8–2.1)	16.9% (8.7–27.5)	8.3% (3.1–17.0)	8	0	7.4 (5.8–12.0)	5.6 (4.7–7.3)	61	47	38.9 (26.6–51.0)	15.0 (7.4–25.1)

NA, Not Available.

### 3.3 The secondary result

**Figure 5:**
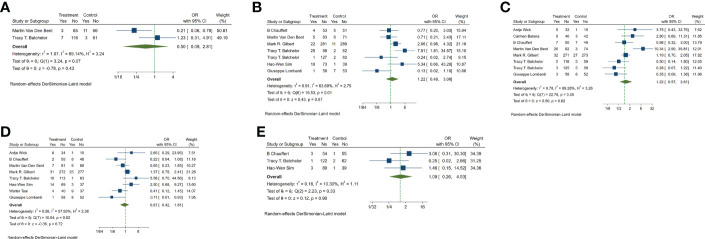
Odds ratio (OR) for adverse events of the included studies. **(A)** leukopenia **(B)** neutropenia **(C)** lymphocytopenia **(D)** thrombocytopenia **(E)** nervous system (mainly headache).

Leukopenia was included in the analysis of the two trials. The results showed that OR=0.50 (0.09-2.81) indicated that the incidence of leukopenia in targeted combined radiotherapy and chemotherapy might be less, but there was no statistical significance (**Figure 5A**). Neutropenia was included in 7 experimental analyses. The results showed that the incidence of neutropenia was higher in OR=1.22 (0.49-3.08) and targeted combined radiotherapy and chemotherapy, but there was no statistical significance (**Figure 5B**). Lymphocytopenia was included in 8 tests, OR=1.22 (0.57-2.61), and the incidence of targeting combined with radiotherapy and chemotherapy may be higher, with no statistical significance(*p*=0.62) (**Figure 5C**). Thrombocytopenia was included in 11 trials, OR=0.87 (0.42-1.81), suggesting that the incidence of thrombocytopenia in combination with radiotherapy and chemotherapy may be less, with no statistical significance (*p*=0.72) (**Figure 5D**). Headache was included in three trials, OR=1.09 (0.26-4.53), with no statistical significance (*p*=0.90) (**Figure 5E**).

### 3.4 Publication bias

**Figure 6:**
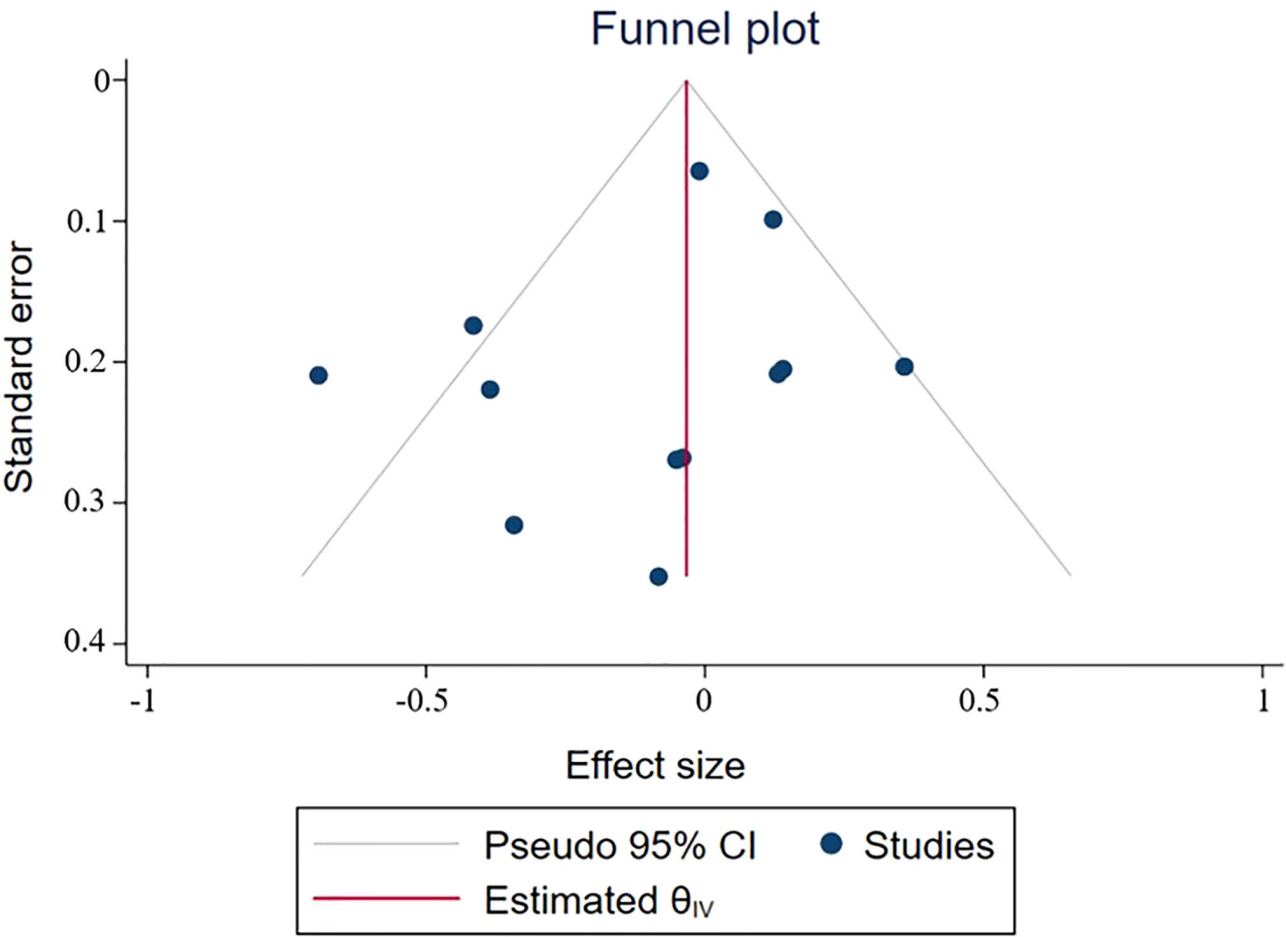
Funnel plot of hazard ratios for overall survival (OS).

**Figure 7:**
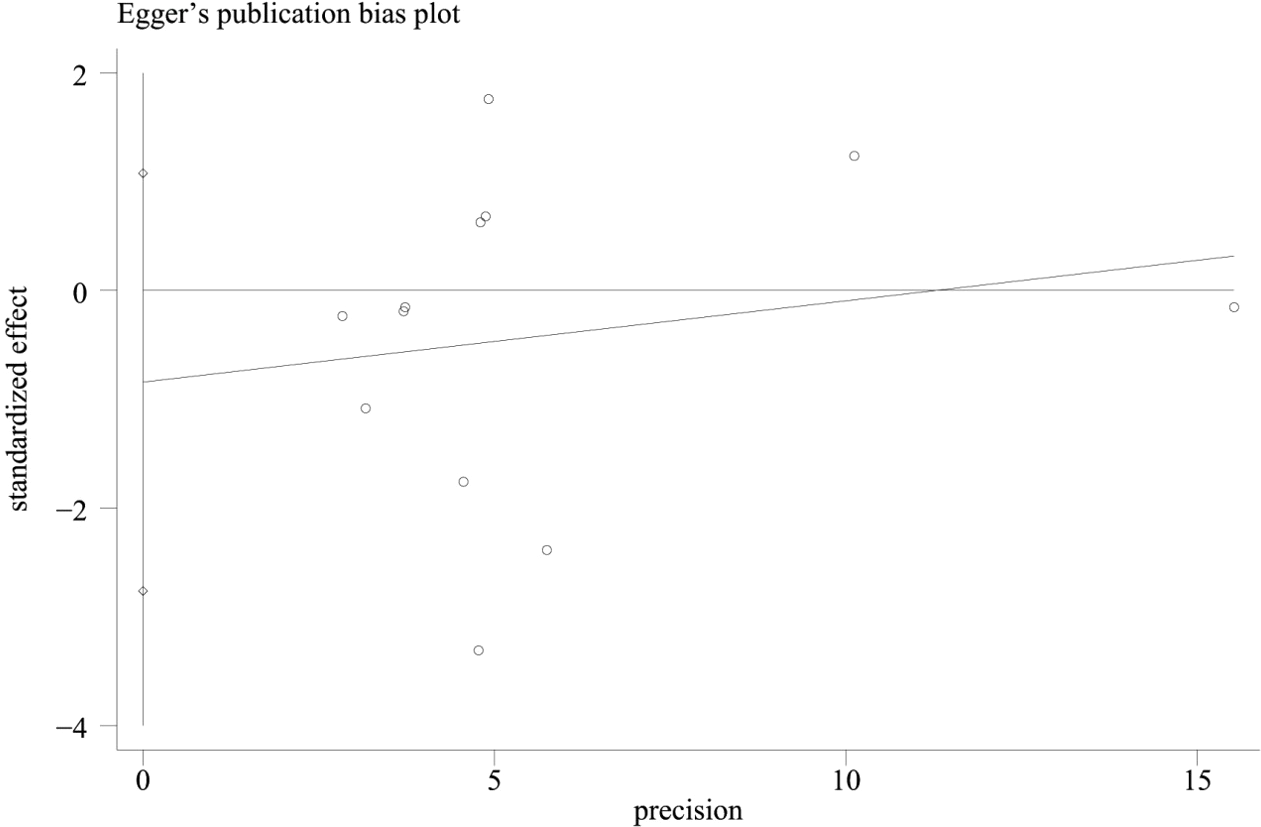
Egger’s publication bias plot of hazard ratios for overall survival (OS).

**Figure 8:**
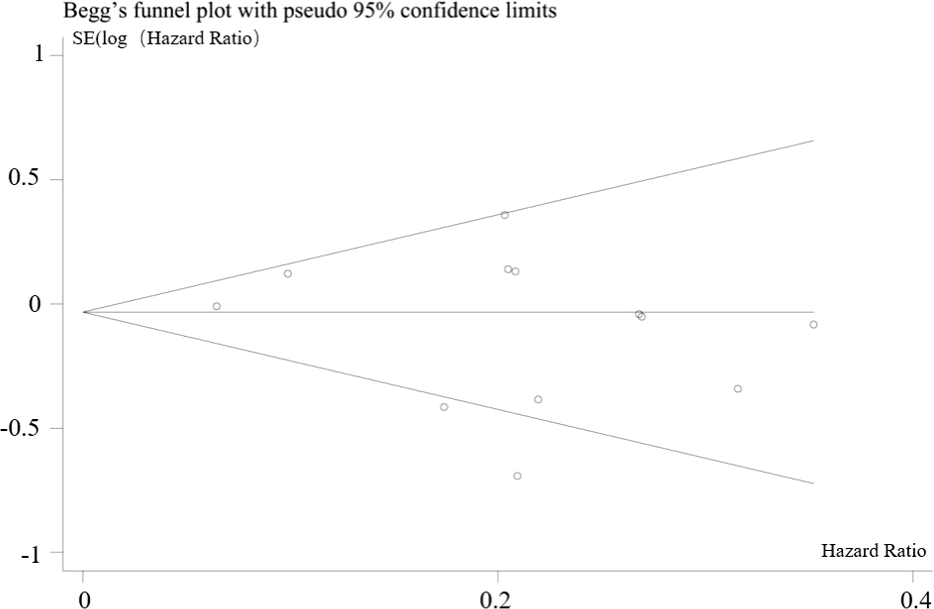
Begg’s funnel plot of hazard ratios for overall survival (OS).

In combination with funnel chart and Egger’s test, *p*=0.352, Begg’s Test, *p* = 0.193, no significant publication bias was found (**Figures 6**–**8**).

### 3.5 Heterogeneity analysis and sensitivity analysis

**Figure 9:**
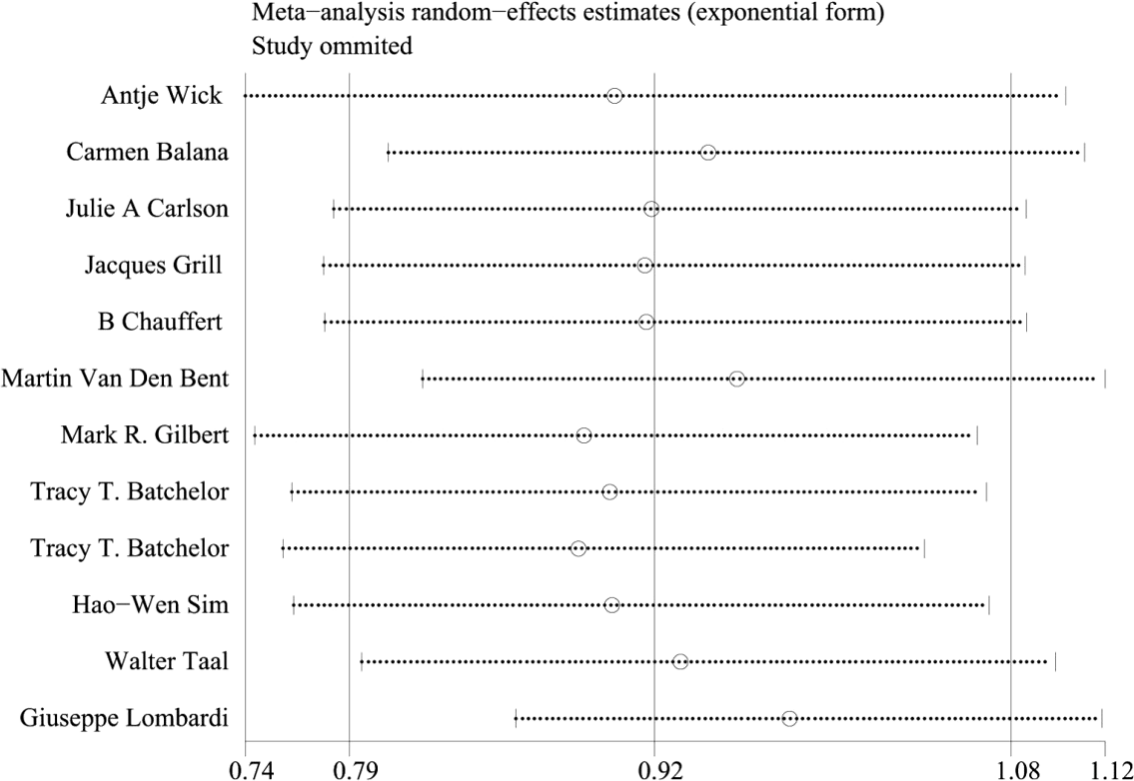
Sensitivity analysis of hazard ratios for overall survival (OS).

Because of the large heterogeneity of PFS (I^2 ^= 95.09%), we only conducted subgroup analysis of it. In OS, the staging of the trial considered by subgroup analysis was one of the sources of heterogeneity of meta-analysis results, and the heterogeneity between Phase II and Phase III trials was greater. The results of Meta regression analysis also confirmed that the staging of the trial was one of the sources of heterogeneity(*p*=0.016). The sensitivity analysis of OS showed good stability (**Figure 9**).

### 3.6 Other

**Figure 10:**
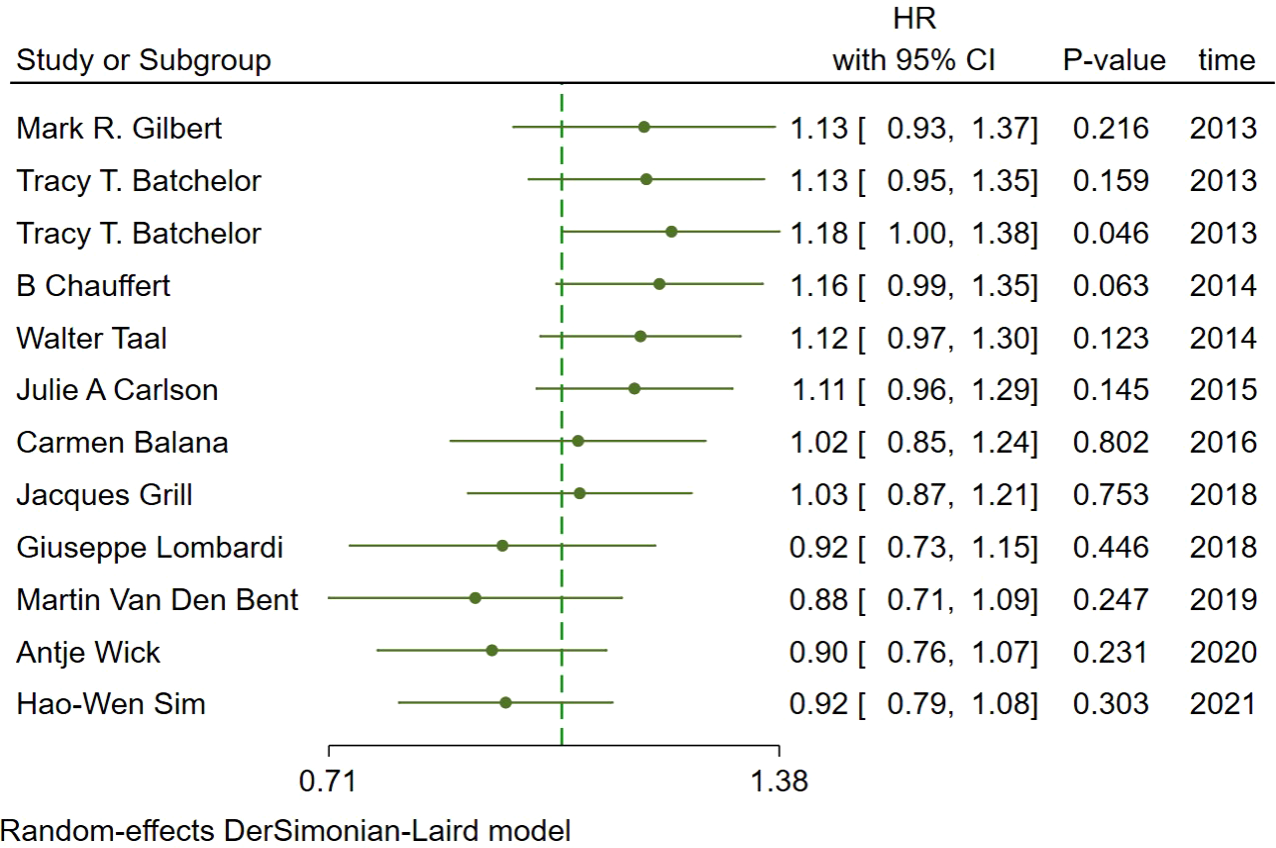
Cumulative meta-analysis of hazard ratios for overall survival (OS).

**Figure 11:**
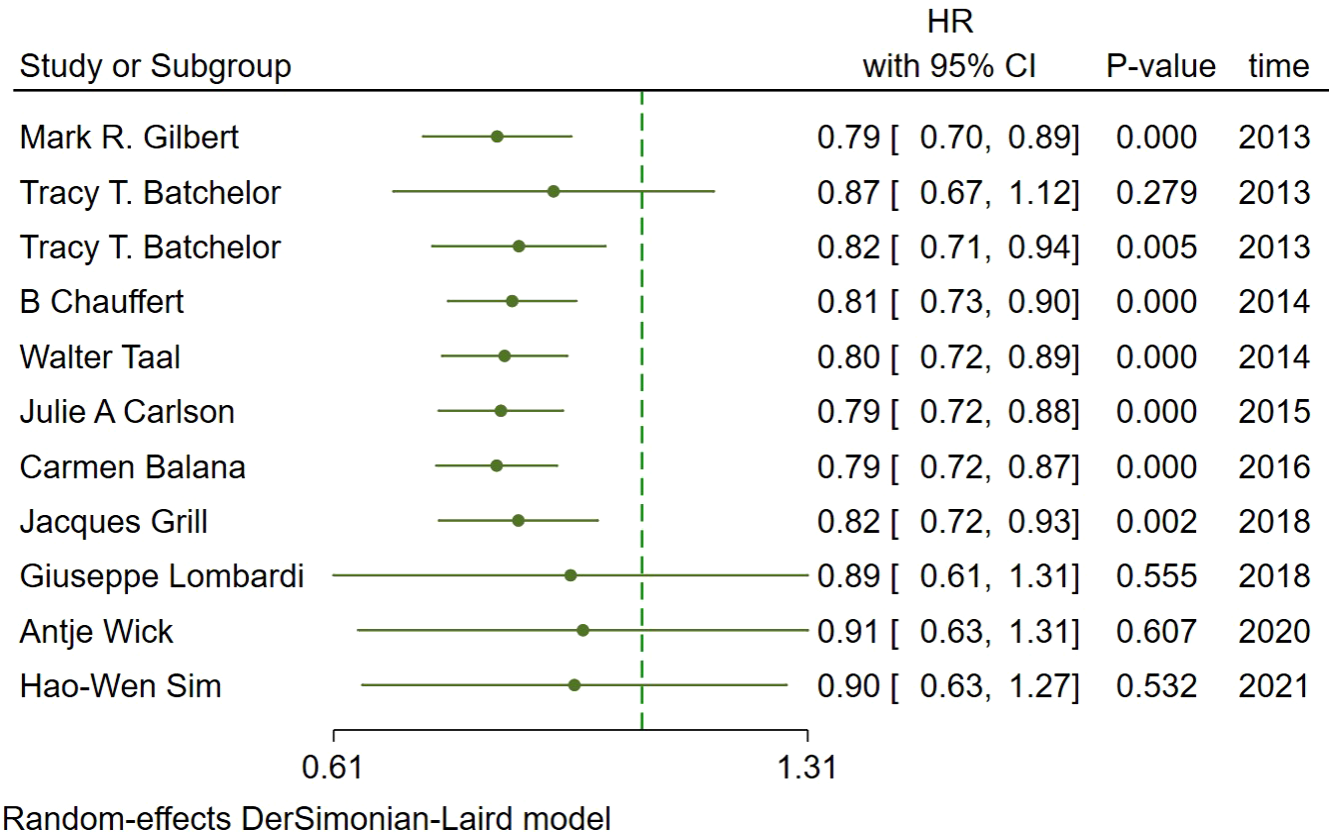
Cumulative meta-analysis of hazard ratios for Progression-free survival (PFS).

In the cumulative meta-analysis, without studying the *p* value, OS showed an overall trend of improvement with the increase of time, while PFS did not show an obvious improvement trend (**Figures 10**, **11**).

## 4 Discussion

Glioblastoma is the most common and invasive primary brain tumor in adults (26). At present, the standard treatment of glioblastoma is maximum surgery, followed by radiotherapy and chemotherapy and temozolomide adjuvant chemotherapy. Despite the active treatment interventions, the survival rate of GBM patients did not improve significantly. Death related recurrence is common in most patients with GBM (4). The basis of chemotherapy is to inhibit the division of rapidly growing cells, which is a feature of cancer cells, but it also affects the rapidly proliferating normal cells, leading to the unique side effects of chemotherapy. The destruction of normal cells, the toxicity of chemotherapy drugs and the development of multidrug resistance support the need to find new effective targeted therapies based on the molecular biological changes of tumor cells. In recent years, targeted therapies have attracted more and more attention. They can induce cancer cell death by blocking biological transduction pathways or specific cancer proteins, or specifically deliver chemotherapy drugs to cancer cells to minimize adverse side effects (27). Targeted therapy has been approved to combine with traditional therapy. In a variety of cancers, combined targeted therapy shows better anti-cancer effect (28–30). Therefore, we studied the efficacy of targeted therapy based on radiotherapy and temozolomide-based chemotherapy.

In the meta-analysis of 12 randomized controlled trials in 1284 glioma patients, there was no statistically significant improvement in OS and PFS of targeted combined radiotherapy and chemotherapy. In phase II trial, compared with radiotherapy and chemotherapy alone, the OS in the targeted combined radiotherapy and chemotherapy group was significantly improved, HR=0.82 (0.67-0.99, *p*=0.038), while in phase III trial, there was no improvement on OS, HR=1.18 (1.00-1.38, *p*=0.046), which was consistent with the results of the study. Martin et al. showed that Depatux-M combined with temozolomide may play a role in recurrent glioblastoma amplified by EGFR, but their findings were not supported by significant evidence in the newly diagnosed glioblastoma in phase III study (20). The results of phase II and phase III trials were different, and the included studies showed that the number of patients in Phase II and Phase II trials was different. The number of patients in phase III trials exceeded 300, which significantly larger than that in phase II trials, and the results of phase III trials may be more reliable. In addition, we speculate that the treatment of glioma patients in phase III trial, including dose and frequency of administration, will also be different from phase II trial, which leads to the difference between the two results. In future clinical trials, researchers should ensure the number of patients as much as possible and use the treatment with the highest consensus at present. Compared with the radiotherapy and chemotherapy alone group, the PFS of the newly diagnosed patients with targeted combined radiotherapy and chemotherapy was statistically improved, HR=0.81 (0.72-0.92) 95% CI, p=0.001. The patients with recurrent GM had HR=0.93 (0.48-1.83) 95% CI, *p*=0.844, which was not statistically significant compared with the radiotherapy and chemotherapy group. We considered that PFS in patients with recurrent GM does not improve due to tolerance to radiotherapy and chemotherapy, increased malignancy of the tumor, or genetic mutations. For example, mutated TP53 is closely associated with poor overall survival in patients with glioblastoma. In addition, TP53 mutation may reduce the chemical sensitivity of glioblastoma to temozolomide by increasing the expression of O (6)-methylguanine-DNA methyltransferase (MGMT) (31). Anaplastic lymphoma kinase (ALK) gene mutation is associated with poor prognosis of glioma and IDH wild type glioblastoma (32). Stefan et al. showed a significant increase in local Met activation in recurrent tumors and confirmed that Met activation may be one of the sources of resistance to EGFR inhibitors and the activated PI3K/mTOR signal pathway may play an important role in glioma recurrence (33). VEGF signal plays an important role in neo-angiogenesis, and its inhibition is a key therapeutic strategy for cancer treatment. VEGF and EGF share common downstream signaling pathways and may function exclusively of one another during oncogenesis and acquired therapeutic resistance (34). Therefore, in order to facilitate analysis, we divided VEGF and EGF receptor inhibitors into a subgroup and compared them with non-VEGF and EGF receptor inhibitors, but there was no significant difference between them. There was no significant increase in the risk of adverse reactions between the targeted combined radiotherapy and chemotherapy group and the radiotherapy and chemotherapy alone group. From the cumulative meta-analysis, we can also see that with the increase of time, the overall OS of patients shows a trend of improvement, indicating that the formulation of drug treatment plan is becoming more and more standardized, and the treatment of patients is becoming more and more effective. This is of great significance for clinical trials of targeted drugs combined with TMZ in the treatment of brain glioma. There are still some shortcomings in this meta-analysis. First, because the HR of some studies (13, 17–19, 24) are not listed in the literature, or the listed HR is hierarchical HR with a large confidence interval, so we use the estimation method described by Tierney to estimate the risk ratio and their 95% confidence interval, which had a certain impact on the accuracy of the study. In future clinical trials, we should encourage researchers to not only display the KM curve, but also list the HR, and point out whether it is univariate HR or hierarchical HR, which will be of great help to analyze targeted glioma treatment. Second, the meta-analysis of PFS has great heterogeneity, and the heterogeneity in subgroup analysis is mainly concentrated in the subgroup of patients with recurrent glioma. In patients with recurrent glioma, the individual condition and treatment plan are more complex, which may lead to heterogeneity. Third, a large part of the statistics of adverse events included in the study are missing data or there are no unified research standards, so it is impossible to systematically analyze the adverse events in each study. It affects our assessment of the safety of targeted drugs combined with radiotherapy and temozolomide-based chemotherapy. In our meta-analysis, in the phase II trials, compared with radiotherapy and chemotherapy of gliomas, targeted drugs combined with temozolomide-based radiotherapy and chemotherapy significantly improved OS, while the OS in the phase III trial did not change. The meta-analysis and systematic review on the efficacy and safety of targeted combined chemotherapy for advanced gastric cancer conducted by Zou et al. also found that for patients with unresectable advanced or recurrent gastric cancer, targeted combined chemotherapy has better overall survival rate and treatment efficiency than traditional chemotherapy (35).

## 5 Conclusion

In this study, we recommend that more attention should be paid to the neuropathological and molecular pathological diagnosis of gliomas in the future treatments and clinical trials. Therefore, there is an urgent need for more basic and clinical trials to explore and evaluate the feasibility of targeted therapy and the corresponding biomarkers, so as to achieve effective personalized treatment choices. Meanwhile, more high-quality randomized controlled trials are needed to provide more and more accurate information. Overall, this meta-analysis showed that there was no significant difference in the incidence of adverse events between the targeted combined radiotherapy and chemotherapy group and the radiotherapy and chemotherapy alone group. In phase II trial, compared with radiotherapy and chemotherapy for glioma, targeted drugs combined with temozolomide-based radiotherapy and chemotherapy could significantly improve OS, while Phase III trial has no improvement in OS. However, the phase III trial was improved. In future clinical trials, researchers should ensure the number of patients as much as possible and use the treatment with the highest consensus at present. Targeting combined with radiotherapy and temozolomide-based chemotherapy can improve PFS in patients with newly diagnosed gliomas. In the treatment of glioma patients, the neuropathological and molecular pathological diagnosis should be improved as much as possible, and the group selection of patients should be refined to achieve the purpose of precise targeted therapy.

## Data availability statement

The original contributions presented in the study are included in the article/supplementary material. Further inquiries can be directed to the corresponding author.

## Author contributions

YM, YW, CN and YL wrote the manuscript. All authors contributed to the article and approved the submitted version.

## References

[1] Ostrom QT Bauchet L Davis FG Deltour I Fisher JL Langer CE . The epidemiology of glioma in adults: A "state of the science" review. Neuro Oncol (2014) 16(7):896–913. doi: 10.1093/neuonc/nou087 24842956 PMC4057143

[2] Jovčevska I . Sequencing the next generation of glioblastomas. Crit Rev Clin Lab Sci (2018) 55(4):264–82. doi: 10.1080/10408363.2018.1462759 29668342

[3] Wirsching HG Galanis E Weller M . Glioblastoma. Handb Clin Neurol (2016) 134:381–97. doi: 10.1016/B978-0-12-802997-8.00023-2 26948367

[4] Touat M Idbaih A Sanson M Ligon KL . Glioblastoma targeted therapy: Updated approaches from recent biological insights. Ann Oncol (2017) 28(7):1457–72. doi: 10.1093/annonc/mdx106 PMC5834086 28863449

[5] Nachbichler SB Schupp G Ballhausen H Niyazi M Belka C . Temozolomide during radiotherapy of glioblastoma multiforme: Daily administration improves survival. Temozolomid zur Strahlentherapie von Glioblastoma multiforme Tägliche Gabe verbessert das Überleben Strahlenther Onkol (2017) 193(11):890–6. doi: 10.1007/s00066-017-1110-4 28197654

[6] Delgado-López PD Corrales-García EM . Survival in glioblastoma: A review on the impact of treatment modalities. Clin Transl Oncol (2016) 18(11):1062–71. doi: 10.1007/s12094-016-1497-x 26960561

[7] Cancer Genome Atlas Research Network . Comprehensive genomic characterization defines human glioblastoma genes and core pathways [published correction appears in nature. Nature (2008) 455(7216):1061–8:. doi: 10.1038/nature07385 PMC2671642 18772890

[8] Shariati M Meric-Bernstam F . Targeting AKT for cancer therapy. Expert Opin Investig Drugs (2019) 28(11):977–88. doi: 10.1080/13543784.2019.1676726 PMC6901085 31594388

[9] Turner NC Slamon DJ Ro J Bondarenko I Im SA Masuda N . Overall survival with palbociclib and fulvestrant in advanced breast cancer. N Engl J Med (2018) 379(20):1926–36. doi: 10.1056/NEJMoa1810527 30345905

[10] Ray-Coquard I Pautier P Pignata S Pérol D González-Martín A Berger R . Olaparib plus bevacizumab as first-line maintenance in ovarian cancer. N Engl J Med (2019) 381(25):2416–28. doi: 10.1056/NEJMoa1911361 31851799

[11] Jänne PA Baik C Su WC Johnson ML Hayashi H Nishio M . Efficacy and safety of patritumab deruxtecan (HER3-DXd) in EGFR inhibitor-resistant, EGFR-mutated non-small cell lung cancer [published correction appears in cancer discov. Cancer Discovery (2022) 12(1):74–89. doi: 10.1158/2159-8290.CD-21-0715 34548309 PMC9401524

[12] Friedman HS Prados MD Wen PY Mikkelsen T Schiff D Abrey LE . Bevacizumab alone and in combination with irinotecan in recurrent glioblastoma. J Clin Oncol (2009) 27(28):4733–40. doi: 10.1200/JCO.2008.19.8721 19720927

[13] Wick A Desjardins A Suarez C Forsyth P Gueorguieva I Burkholder T . Phase 1b/2a study of galunisertib, a small molecule inhibitor of transforming growth factor-beta receptor I, in combination with standard temozolomide-based radiochemotherapy in patients with newly diagnosed malignant glioma. Invest New Drugs (2020) 38(5):1570–9. doi: 10.1007/s10637-020-00910-9 PMC7497674 32140889

[14] Robins HI Zhang P Gilbert MR Chakravarti A de Groot JF Grimm SA . A randomized phase I/II study of ABT-888 in combination with temozolomide in recurrent temozolomide resistant glioblastoma: An NRG oncology RTOG group study. J Neurooncol (2016) 126(2):309–16. doi: 10.1007/s11060-015-1966-z PMC4720526 26508094

[15] Tierney JF Stewart LA Ghersi D Burdett S Sydes MR . Practical methods for incorporating summary time-to-event data into meta-analysis. Trials (2007) 8:16. doi: 10.1186/1745-6215-8-16 17555582 PMC1920534

[16] Balana C De Las Penas R Sepúlveda JM Gil-Gil MJ Luque R Gallego O . Bevacizumab and temozolomide versus temozolomide alone as neoadjuvant treatment in unresected glioblastoma: The GENOM 009 randomized phase II trial. J Neurooncol (2016) 127(3):569–79. doi: 10.1007/s11060-016-2065-5 26847813

[17] Carlson JA Reddy K Gaspar LE Ney D Kavanagh BD Damek D . Hypofractionated-intensity modulated radiotherapy (hypo-IMRT) and temozolomide (TMZ) with or without bevacizumab (BEV) for newly diagnosed glioblastoma multiforme (GBM): a comparison of two prospective phase II trials. J Neurooncol (2015) 123(2):251–7. doi: 10.1007/s11060-015-1791-4 25920710

[18] Grill J Massimino M Bouffet E Azizi AA McCowage G Cañete A . Open-label, randomized, multicenter trial (HERBY) of bevacizumab in pediatric patients with newly diagnosed high-grade glioma. J Clin Oncol (2018) 36(10):951–8. doi: 10.1200/JCO.2017.76.0611 29412784

[19] Chauffert B Feuvret L Bonnetain F Taillandier L Frappaz D Taillia H . Randomized phase II trial of irinotecan and bevacizumab as neo-adjuvant and adjuvant to temozolomide-based chemoradiation compared with temozolomide-chemoradiation for unresectable glioblastoma: final results of the TEMAVIR study from ANOCEF†. Ann Oncol (2014) 25(7):1442–7. doi: 10.1093/annonc/mdu148 24723487

[20] Van Den Bent M Eoli M Sepulveda JM Smits M Walenkamp A Frenel JS . INTELLANCE 2/EORTC 1410 randomized phase II study of depatux-m alone and with temozolomide vs temozolomide or lomustine in recurrent EGFR amplified glioblastoma. Neuro Oncol (2020) 22(5):684–93. doi: 10.1093/neuonc/noz222 PMC7229258 31747009

[21] Gilbert MR Dignam JJ Armstrong TS Wefel JS Blumenthal DT Vogelbaum MA . A randomized trial of bevacizumab for newly diagnosed glioblastoma. N Engl J Med (2014) 370(8):699–708. doi: 10.1056/NEJMoa1308573 24552317 PMC4201043

[22] Batchelor TT Mulholland P Neyns B Nabors LB Campone M Wick A . Phase III randomized trial comparing the efficacy of cediranib as monotherapy, and in combination with lomustine, versus lomustine alone in patients with recurrent glioblastoma. J Clin Oncol (2013) 31(26):3212–8. doi: 10.1200/JCO.2012.47.2464 PMC4021043 23940216

[23] Sim HW McDonald KL Lwin Z Barnes EH Rosenthal M Foote MC . A randomized phase II trial of veliparib, radiotherapy, and temozolomide in patients with unmethylated MGMT glioblastoma: The VERTU study. Neuro Oncol (2021) 23(10):1736–49. doi: 10.1093/neuonc/noab111 PMC8485443 33984151

[24] Taal W Oosterkamp HM Walenkamp AM Dubbink HJ Beerepoot LV Hanse MC . Single-agent bevacizumab or lomustine versus a combination of bevacizumab plus lomustine in patients with recurrent glioblastoma (BELOB trial): A randomised controlled phase 2 trial. Lancet Oncol (2014) 15(9):943–53. doi: 10.1016/S1470-2045(14)70314-6 25035291

[25] Lombardi G De Salvo GL Brandes AA Eoli M Rudà R Faedi M . Regorafenib compared with lomustine in patients with relapsed glioblastoma (REGOMA): A multicentre, open-label, randomised, controlled, phase 2 trial. Lancet Oncol (2019) 20(1):110–9. doi: 10.1016/S1470-2045(18)30675-2 30522967

[26] Tan AC Ashley DM López GY Malinzak M Friedman HS Khasraw M . Management of glioblastoma: State of the art and future directions. CA Cancer J Clin (2020) 70(4):299–312. doi: 10.3322/caac.21613 32478924

[27] Pérez-Herrero E Fernández-Medarde A . Advanced targeted therapies in cancer: Drug nanocarriers, the future of chemotherapy. Eur J Pharm Biopharm (2015) 93:52–79. doi: 10.1016/j.ejpb.2015.03.018 25813885

[28] Upadhya A Yadav KS Misra A . Targeted drug therapy in non-small cell lung cancer: Clinical significance and possible solutions-part I. Expert Opin Drug Deliv (2021) 18(1):73–102. doi: 10.1080/17425247.2021.1825377 32954834

[29] Liu JF Barry WT Birrer M Lee JM Buckanovich RJ Fleming GF . Combination cediranib and olaparib versus olaparib alone for women with recurrent platinum-sensitive ovarian cancer: a randomised phase 2 study. Lancet Oncol (2014) 15(11):1207–14. doi: 10.1016/S1470-2045(14)70391-2 PMC4294183 25218906

[30] Loibl S O'Shaughnessy J Untch M Sikov WM Rugo HS McKee MD . Addition of the PARP inhibitor veliparib plus carboplatin or carboplatin alone to standard neoadjuvant chemotherapy in triple-negative breast cancer (BrighTNess): A randomised, phase 3 trial. Lancet Oncol (2018) 19(4):497–509. doi: 10.1016/S1470-2045(18)30111-6 29501363

[31] Wang X Chen JX Liu JP You C Liu YH Mao Q . Gain of function of mutant TP53 in glioblastoma: Prognosis and response to temozolomide. Ann Surg Oncol (2014) 21(4):1337–44. doi: 10.1245/s10434-013-3380-0 24248532

[32] Bu L Hameed NUF Luo C Hong P Zhou X Wang S . Germline ALK variations are associated with a poor prognosis in glioma and IDH-wildtype glioblastoma. J Neurooncol (2021) 152(1):27–36. doi: 10.1007/s11060-020-03676-5 33486679

[33] Klingler S Guo B Yao J Yan H Zhang L Vaseva AV . Development of resistance to EGFR-targeted therapy in malignant glioma can occur through EGFR-dependent and -independent mechanisms. Cancer Res (2015) 75(10):2109–19. doi: 10.1158/0008-5472.CAN-14-3122 PMC4433602 25808866

[34] Le X Nilsson M Goldman J Reck M Nakagawa K Kato T . Dual EGFR-VEGF pathway inhibition: A promising strategy for patients with EGFR-mutant NSCLC. J Thorac Oncol (2021) 16(2):205–15. doi: 10.1016/j.jtho.2020.10.006 33096270

[35] Zou K Yang S Zheng L Yang C Xiong B . Efficacy and safety of target combined chemotherapy in advanced gastric cancer: A meta-analysis and system review. BMC Cancer (2016) 16(1):737. doi: 10.1186/s12885-016-2772-5 27633381 PMC5025570

